# MAPK1 Mediates MAM Disruption and Mitochondrial Dysfunction in Diabetic Kidney Disease via the PACS-2-Dependent Mechanism

**DOI:** 10.7150/ijbs.89291

**Published:** 2024-01-01

**Authors:** Shanshan Liu, Shuai Han, Cuili Wang, Hongjun Chen, Qiannan Xu, Shi Feng, Yucheng Wang, Jihong Yao, Qin Zhou, Xuanli Tang, Li Lin, Lidan Hu, Alan J Davidson, Bing Yang, Cunqi Ye, Fan Yang, Jianhua Mao, Chao Tong, Jianghua Chen, Hong Jiang

**Affiliations:** 1 Kidney Disease Center, the First Affiliated Hospital, College of Medicine, Zhejiang University, Hangzhou, China.; 2 Key Laboratory of Kidney Disease Prevention and Control Technology, Hangzhou, China.; 3 Institute of Nephrology, Zhejiang University, Hangzhou, China.; 4 Zhejiang Clinical Research Center of Kidney and Urinary System Disease, Hangzhou, China.; 5 Department of nephrology, Hangzhou TCM Hospital Affiliated to Zhejiang Chinese Medical University, Hangzhou, China.; 6 Department of Nephrology, The Children's Hospital, Zhejiang University School of Medicine, National Clinical Research Center for Child Health, Hangzhou, China.; 7 Department of Molecular Medicine & Pathology, School of Medical Sciences, Faculty of Medical & Health Sciences, The University of Auckland, Auckland, New Zealand.; 8 Zhejiang Provincial Key Laboratory for Cancer Molecular Cell Biology, Life Sciences Institute, Zhejiang University, Hangzhou, China.; 9 Department of Biophysics, and Kidney Disease Center of the First Affiliated Hospital, Zhejiang University School of Medicine, Hangzhou, China.; 10 MOE Key Laboratory for Biosystems Homeostasis & Protection and Innovation Center for Cell Signaling Network, Life Sciences Institute, Zhejiang University, Hangzhou, China.

**Keywords:** diabetic kidney disease, mitochondria, mitochondria-associated endoplasmic reticulum membrane, MAPK1, PACS-2

## Abstract

Diabetic kidney disease (DKD) is a leading cause of end-stage renal disease (ESRD). Mitochondrial dysfunction in renal tubules, occurring early in the disease, is linked to the development of DKD, although the underlying pathways remain unclear. Here, we examine diabetic human and mouse kidneys, and HK-2 cells exposed to high glucose, to show that high glucose disrupts mitochondria-associated endoplasmic reticulum membrane (MAM) and causes mitochondrial fragmentation. We find that high glucose conditions increase mitogen-activated protein kinase 1(MAPK1), a member of the MAP kinase signal transduction pathway, which in turn lowers the level of phosphofurin acidic cluster sorting protein 2 (PACS-2), a key component of MAM that tethers mitochondria to the ER. MAPK1-induced disruption of MAM leads to mitochondrial fragmentation but this can be rescued in HK-2 cells by increasing PACS-2 levels. Functional studies in diabetic mice show that inhibition of MAPK1 increases PACS-2 and protects against the loss of MAM and the mitochondrial fragmentation. Taken together, these results identify the MAPK1-PACS-2 axis as a key pathway to therapeutically target as well as provide new insights into the pathogenesis of DKD.

## Introduction

Diabetic kidney disease (DKD) is one of the most common microvascular complications of diabetes mellitus and is a major cause of end-stage renal disease (ESRD) 1, 2. Approximately 50% of patients with DKD progress to ESRD in developed countries, leading to major societal and medical burdens 3, 4. However, the pathogenic mechanisms underlying DKD remain unclear.

Mitochondria are highly abundant in the kidney [5, 6]and are mainly present in renal tubular cells 7. Under physiological conditions, mitochondria ensure efficient oxidative phosphorylation and ATP production in cells. When cells are in a stressful environment, mitochondrial fragmentation and dysfunction result in impaired energy metabolism 8, 9. Mitochondrial dysfunction occurs in DKD before histological and laboratory signs and may be responsible for its development 10, 11. Thus, protection against mitochondrial dysfunction may not only prevent DKD progression but also reduce the risk of ESRD 12-14.

Mitochondria can directly interact with the endoplasmic reticulum (ER) to form 'mitochondria-associated endoplasmic reticulum membrane' (MAM), which are involed in regulation of multiple intracellular events, including the control of lipid biosynthesis, Ca^2+^ homeostasis, mitochondrial function, and ER stress 15-17. Disruption of MAM integrity was related to the development of many diseases, including obesity 18, diabetes 19 and neurodegenerative disorders 20. Studies have shown that MAM is a critical hub for glucose homeostasis and that its disruption can induce mitochondrial dysfunction, which contribute to insulin resistance under diabetic conditions 19, 21.

MAM is a dynamic connection composed of a series of proteins with various functions. The disruption of MAM proteins may impair their structure and function 15, 20. A comprehensive proteomic analysis revealed that 144 proteins found in MAM were altered under high glucose conditions 22. As the first identified MAM-resident protein, phosphofurin acidic cluster sorting protein 2 (PACS-2) is a key regulator of MAM and has been reported to have wide-ranging effects on metabolic diseases, such as obesity and insulin resistance 23, 24. Recent studies have found that PACS-2 is mainly expressed in renal tubules, and its expression is down-regulated by high glucose. Knockdown of *Pacs-2* causes disruption of MAM integrity and mitochondrial dysfunction in the renal tubules of diabetic mice 25, suggesting that PACS-2 plays a key role in maintaining the stability of MAM in DKD. However, the mechanisms underlying high glucose-induced PACS-2 loss in DKD remain unclear.

Mitogen-activated protein kinase 1 (MAPK1) is an important component of the MAPK pathway that can be activated by various stimuli (cytokines, growth factors, reactive oxygen species, and high glucose) and regulates cell differentiation, proliferation, growth, and death 26, 27. Activation of MAPK1 leads to mitochondrial dysfunction, such as mitochondrial fragmentation, fission, or fusion, and disruption of mitochondrial homeostasis, as observed in cancer 28, neuronal apoptosis 29, and chondrocyte apoptosis 30. Under diabetic conditions, high glucose can activate the MAPK signaling pathway and cause tubulointerstitial injury in DKD, which can be improved by inhibiting MAPK activation 31-33. However, the mechanisms of MAPK1-mediated DKD remain unclear and need to be further investigated.

In this study, we examine MAM and mitochondria in diabetic human and mouse kidney tissues as well as HK-2 cells grown under high glucose conditions. We find that high glucose conditions are associated with a profound disruption in MAM and mitochondria integrity. These effects were attributed to increased levels of MAPK1, resulting in a loss of PACS-2 which is required to maintain MAM and prevent mitochondrial fragmentation. This work identifies MAPK1 as a key driver of MAM disruption and mitochondrial dysfunction in diabetes and uncovers the MAPK1-PACS-2 axis as a target for developing new therapies for DKD.

## Materials and methods

### Renal biopsy samples from patients with DKD

Forty renal biopsy samples from patients with DKD were used in this study, including 20 cases of DKD I-II and 20 cases of DKD III-IV. Twenty matched donor kidney samples were used as healthy controls. **Table S1** presents the clinical characteristics of enrolled patients and controls. All renal biopsy tissue specimens were obtained from the First Affiliated Hospital, College of Medicine, Zhejiang University, between 2012 and 2021. The Ethics Committee of the First Affiliated Hospital, College of Medicine, Zhejiang University approved the use of human renal tissue in this study (#2019-338).

### Mouse model of DKD

Six-week-old male C57BL/6J mice and seven-week-old male db/db mice were purchased from the Zhejiang University Animal Center (Hangzhou, China) and Sai Ye Biotechnology Co., Ltd. (Suzhou, China) respectively. All mice were housed under controlled environmental conditions (temperature 22°C, 12-hour darkness period). Type-1 diabetes was induced in C57BL/6J mice by intraperitoneal injection (i.p.) with STZ (S0130, Sigma-Aldrich) at 50 mg/kg body weight in 0.1 mmol/l citrate buffer once each day for 5 continuous days at 6 weeks of age, whereas control mice received 0.1 mmol/l fresh citrate buffer daily for 5 days, as previously described 34. The db/db mice were sacrificed after two months of high-fat diet. To examine the regulatory role of MAPK1 in DKD, diabetic and non-diabetic mice were randomly assigned to four groups and treated with or without the MAPK1 inhibitor VX-11e (S7709, Selleck) at an optimal dose of 5 mg/kg i.p. twice a week for 20 weeks.

Body weight and fasting blood glucose levels (6 h) were determined weekly for the first two weeks and every two weeks thereafter. Mice with blood glucose levels ≥16.7 mmol/l at 2 weeks after the last STZ injection were used. All the mice were euthanized 20 weeks after the last STZ injection. The Ethics Committee of the First Affiliated Hospital, College of Medicine, Zhejiang University, approved the animal experimental protocols (#2019-461).

### Cell cultures

A human renal proximal tubular epithelial cell line (HK-2) was obtained from the American Type Culture Collection (ATCC) and cultured in Dulbecco's Modified Eagle Medium containing 5 ng/mL epidermal growth factor (EGF) (PHG0311, Gibco), 5% fetal bovine serum (10091-148, Gibco), 100 U/mL penicillin and 100 μg/mL streptomycin (P1400, Solarbio) under low glucose (LG, 5mM D-glucose) or high glucose (HG, 30mM D-glucose) conditions for 24h. To knock down *MAPK1* or *PACS-2*, HK-2 cells were transfected with *MAPK1* and/or *PACS-2* siRNA obtained from Hanbio Biotechnology (Shanghai, China) using RNAiMAX (13778150, Invitrogen). In addition, HK-2 cells stably expressing MAPK1 were established by transfecting the cells with the PC-h-MAPK1 plasmid (Hanbio Biotechnology) using Lipofectamine 3000 (L3000015, Invitrogen) and selected with puromycin, while HK-2 cells stably expressing PACS-2 were established by infecting HK-2 cells with lentivirus (pcSLenti-EF1-BSR-CMV-PACS2-3xFLAG-WPRE) obtained from ObiO Technology Corp., Ltd., and then selected with blasticidin S.

### Measurement of urine albumin/creatinine ratio

Urine albumin was tested using a mouse albumin ELISA kit (E99-134, Bethyl) and urine creatinine was detected by a creatinine parameter assay kit (KGE005, R&D systems) following the manufacturer's instructions.

### RNA sequencing

RNA samples from the kidney cortex of diabetic and control mice were prepared using the TruSeq RNA Sample Preparation Kit. Briefly, purification of poly-A-containing mRNA molecules from 3 μg of total RNA was reverse transcribed into cDNA, and cDNA fragments were purified, end blunted, 'A' tailed, and adaptor ligated. Then, DNA with adaptor molecules was selective enriched and amplificated by PCR. Libraries were sequenced on the HiSeq 2500 platform following identification with the Agilent 2100 Bioanalyzer, as well as quantification using Qubit and qPCR.

We used Bowtie1 software to map sequencing reads onto reference gene sets, and Perl scripts to process the mapping results to generate gene expression profiles.

### Immunohistochemistry and immunofluorescence

Formalin-fixed paraffin-embedded sections (2 μm) were used for hematoxylin-eosin (HE), periodic acid schiff (PAS), masson staining, immunohistochemistry (IHC), and immunofluorescence (IF) staining 35, 36. A semiquantitaive histologic scoring was performed to evaluate the degree of glomeruli sclerosis and tubular interstitial damage as before 37. The antibodies used for IHC were MFN2 (9482, CST), DRP1 (ab184247, Abcam), MAPK1 (ab32081, Abcam), and PACS-2 (19508-1-AP, Proteintech). The antibodies used for IF were Calnexin (10427-2-AP, Proteintech), MAPK1 (sc-1647, Santa Cruz Biotechnology), PACS-2 (19508-1-AP, Proteintech), DRP1 (8570, CST), TOM20 (HPA011562, Sigma-Aldrich) and DAPI (D1306, Invitrogen). At least three fields of each section were randomly acquired using a Leica DM4000 microscope and a Nikon A1 confocal microscope and analyzed using ImageJ software.

### Western blot analysis

Total proteins from kidney cortex or HK-2 cells were extracted by RIPA buffer with protease and phosphatase inhibitors. Targeted proteins were tested by specific antibodies: MFN2 (9482, CST), DRP1 (8570, CST), MAPK1 (ab32081, Abcam), PACS-2 (19508-1-AP, Proteintech), Calnexin (10427-2-AP, Proteintech), Cox IV (11967S, CST), and FACL4 (22401-1-AP, Proteintech). Target proteins were visualized using a chemiluminescent substrate and analyzed using Image Lab software.

### Transmission electron microscopy (TEM)

Renal biopsy tissues or cultured HK-2 cells were successively fixed with 2.5% glutaraldehyde, 1% osmium tetroxide, stained with 2% uranyl acetate, gradually dehydrated with ethanol and acetone, embedded in Eponate resin, and polymerized. Ultrathin sections (80-90 nm) stained were photographed using a transmission electron microscope (TecnaiG2 Spirit TWIN). To assess MAM length, we measued the mitochondrial membrane in contact with the ER within a 10-30 nm range and normalized to the mitochondrial perimeter 38. To quantify fragmented mitochondria, we used ImageJ to measure the cross-sectional area of each mitochondrion and sorted mitochondria of different sizes to reveal their distribution 39.

### In situ proximity ligation assay (PLA)

The proximity of VDAC1 and IP3R1 in the paraffin-embedded kidney sections was measured and quantified using an in situ PLA assay kit (DUO92012, Sigma-Aldrich) to reflect MAM. Briefly, after deparaffinization, hydration, antigen retrieval, and blocking, renal tissues were incubated overnight at 4℃ with anti-VDAC1 antibody (66345-1-Ig, Proteintech) and anti-IP3R1 antibody (19962-1-AP, Proteintech). Then, the sections were incubated with PLA probe followed by polymerase. After washing, the sections were incubated with substrate solution, stained with hematoxylin, and examined under a light microscope.

### Purification of MAM

Percoll gradient fractionation was used to purify MAM 40. Briefly, 100 mg of the kidney cortex was harvested in IB-1 (225 mM mannitol, 75 mM sucrose, 0.5% BSA, 0.5 mM EGTA, and 30 mM Tris-HCl, pH 7.4) and then washed 3-4 times in IB-3 (225 mM mannitol, 75 mM sucrose, and 30 mM Tris-HCl, pH 7.4). Samples were lysed using tissue disruptors from IB-1 supplemented with protease inhibitors. Homogenates were centrifuged twice at 740 × g for 5 min at 4℃ and collected supernatants were centrifuged at 9,000g for 10 min at 4℃. The resulting pellet was saved, gently resuspended in 10 mL of ice-cold IB-2 (225-mM mannitol, 75-mM sucrose, 0.5% BSA, and 30-mM Tris-HCl, pH 7.4), and then centrifuged at 10,000g for 10 min at 4℃. The crude mitochondrial pellet was gently resuspended in 10 mL IB-3 and centrifuged again at 10,000g for 10 min at 4℃, subsequently resuspended in 2 mL ice-cold MRB (250 mM mannitol, 5 mM HEPES (pH 7.4), and 0.5 mM EGTA). Then, the crude mitochondrial fraction was layered onto a 30% Percoll gradient (Percoll medium:225 mM mannitol, 25 mM HEPES (pH 7.4), 1 mM EGTA, and 30% Percoll (vol/vol)) and centrifuged at 95,000 × g for 30 min at 4℃ using a Beckman ultracentrifuge in an SW41ti rotor. Pure mitochondrial fractions were obtained from the bottom of the tube with MAM localized in the intermediate layer. MAM and mitochondria were collected and diluted 10-fold with MRB. MAM was centrifuged at 100,000 g for 60 min while mitochondria were centrifuged at 6,300g for 10 min at 4℃. All MAM and mitochondrial pellets were resuspended in small amounts of MRB.

### Plasmid reporter transfection

Plasmids (pLVX-Mitot-spGFP11×2, plx304-spGFP1-10-Ert) were gifts from Chao Tong (Zhejiang University) and served as a genetically encoded reporter using the split GFP protein for specific labeling of MAM 41. When the distance between the mitochondria and the ER is approximately 10-30 nm, spGFP1-10 and spGFP11 refold and emit green fluorescence. The green fluorescence emitted by cells simultaneously expressing the two plasmids indicates the position of MAM. HK-2 cells were transfected with both plasmids using the lipo2000 transfection reagent (11668019, ThermoFisher Scientific) following the manufacturer's recommendations. Then, HK-2 cells were fixed and incubed with anti-TOM20 (red) antibody (HPA011562, Sigma-Aldrich) to label mitochondria. Images were acquired using confocal microscopy, and the areas of MAM (green fluorescence) and mitochondria (red fluorescence) were measured in each cell by imageJ. The size of MAM was calculated using the ratio of the area of MAM to the area of the mitochondria.

### Immunoprecipitation

HK-2 cells that stably expressed MAPK1 were lysed with immunoprecipitation lysis buffer (R0030, Solarbio) supplemented with protease inhibitors and incubated overnight at 4°C with anti-PACS-2 antibody (19508-1-AP, Proteintech), then incubated with Protein A/G mag beads (L00277, Genscript) for 5 h at 4°C, and the beads-protein complexes were collected for Western blot analysis to detect MAPK1 expression (Sc-1647, Santa Cruz Biotechnology).

### Statistical analyses

Statistical analyses were performed using SPSS and GraphPad Prism. Normally distributed data are presented as mean ± SD and t-test was performed to compare the results between two groups. Non-normally distributed data are presented as medians (interquartile ranges) and were analyzed using non-parametric tests. The correlation between two numerical variables was determined by pearson's correlation analysis and sex comparisons between groups were performed using the χ2 test. Differences were considered statistically significant at *P* < 0.05.

## Results

### Disruption of MAM integrity and mitochondrial dysfunction in renal tubules of patients with DKD, STZ-induced diabetic mice, and high glucose-stimulated HK-2 cells

We first observed at least 1000 mitochondria in the tubular cells of healthy controls using TEM and found that 37.64% contained MAM, 2.57% were undergoing fusion or fission and 1.05% were undergoing mitophagy (**Figure S1A-B**). Similar results were also found in tubular cells of control mice and cultured HK-2 cells exposed to low glucose (**Figure S1C-F**). Thus, mitochondria involved in MAM accounted for a relatively large proportion of the total mitochondria in renal tubular cells, suggesting that MAM may play a crucial role in maintaining mitochondrial homeostasis.

**Figure 1:**
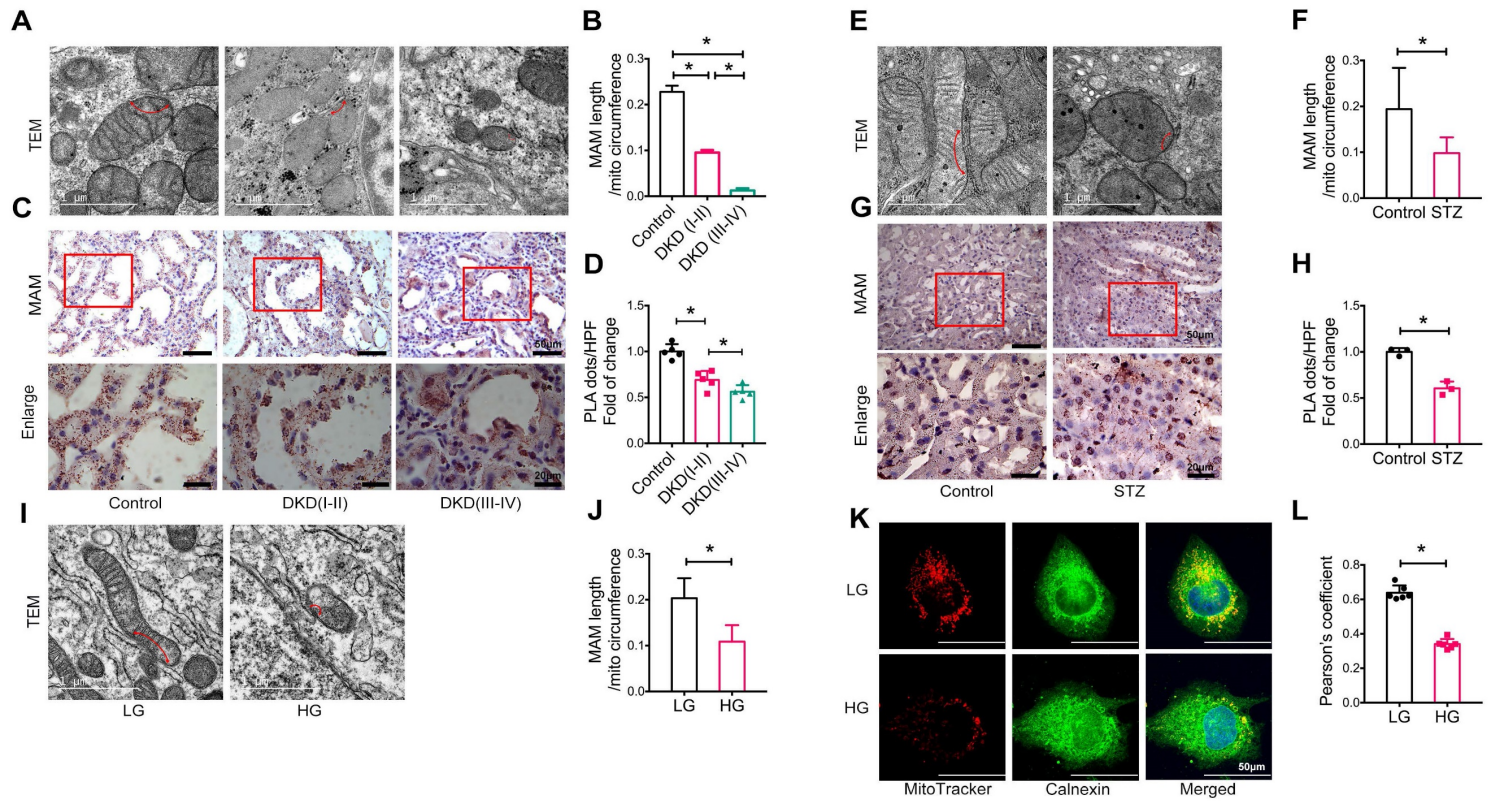
** Disruption of MAM integrity in renal tubules of patients with DKD, diabetic mice, and high glucose stimulated HK-2 cells.** (A) TEM analysis of the length of MAM in renal tubules of healthy controls and patients with DKD. Red arrows indicate MAM regions (Scale bar, 1μm). (B) Quantification of MAM length in different groups. At least 600 MAM in each group were analyzed. (C) Number of MAM detected by in-situ PLA in tubular cells of patients with DKD and healthy controls (Scale bar, 50/20μm). (D) Relative number of PLA dots/ HPF of different groups, n=5. (E) TEM analysis of the length of MAM in renal tubules of STZ-induced diabetic mice and controls (Scale bar, 1μm). (F) Quantification of MAM length in different groups, at least 80 MAM in each group were analyzed. (G) Number of MAM detected by in-situ PLA in tubular cells of STZ-induced diabetic mice and controls (Scale bar, 50/20μm). (H) Relative number of PLA dots/HPF of diabetic and control mice, n=3. (I) TEM analysis of the length of MAM in HK-2 cells exposed to high/low glucose (Scale bar, 1μm). (J) Quantification of MAM length in different groups in HK-2 cells, at least 10 MAM in each group were analyzed. (K) Immunostaining of mitochondria (MitoTracker, red) and ER (Calnexin, green) (Scale bar, 50μm). (L) Pearson's correlation coefficient of mitochondria and ER of different groups, n=6. Data are the mean ± SD, * *P* < 0.05.

We next examined the integrity of MAM in patients and animal models of DKD and HK-2 cells treated with high glucose using TEM, PLA, and immunofluorescence. Compared to healthy controls (approximately 22.79% of the mitochondrial perimeter), TEM revealed a marked decrease in the length of MAM in the renal tubules of patients with DKD (I-II) (approximately 9.53% of the mitochondrial perimeter), which became much shorter (approximately 1.26% of the mitochondrial perimeter) in patients with DKD (III-IV) (**Figure 1A-B**). Analysis using in situ PLA confirmed these hypotheses (**Figure 1C-D**). In STZ-induced diabetic mice, all of which developed hyperglycemia with DKD, including a significant increase in albuminuria and progressive renal injury (**Figure S2**), both TEM and PLA clearly detected a disruption of MAM integrity (**Figure 1E-H**). This was also observed in db/db mice (**Figure S3**) and HK-2 cells exposed to high glucose (**Figure 1I-L**).

**Figure 2:**
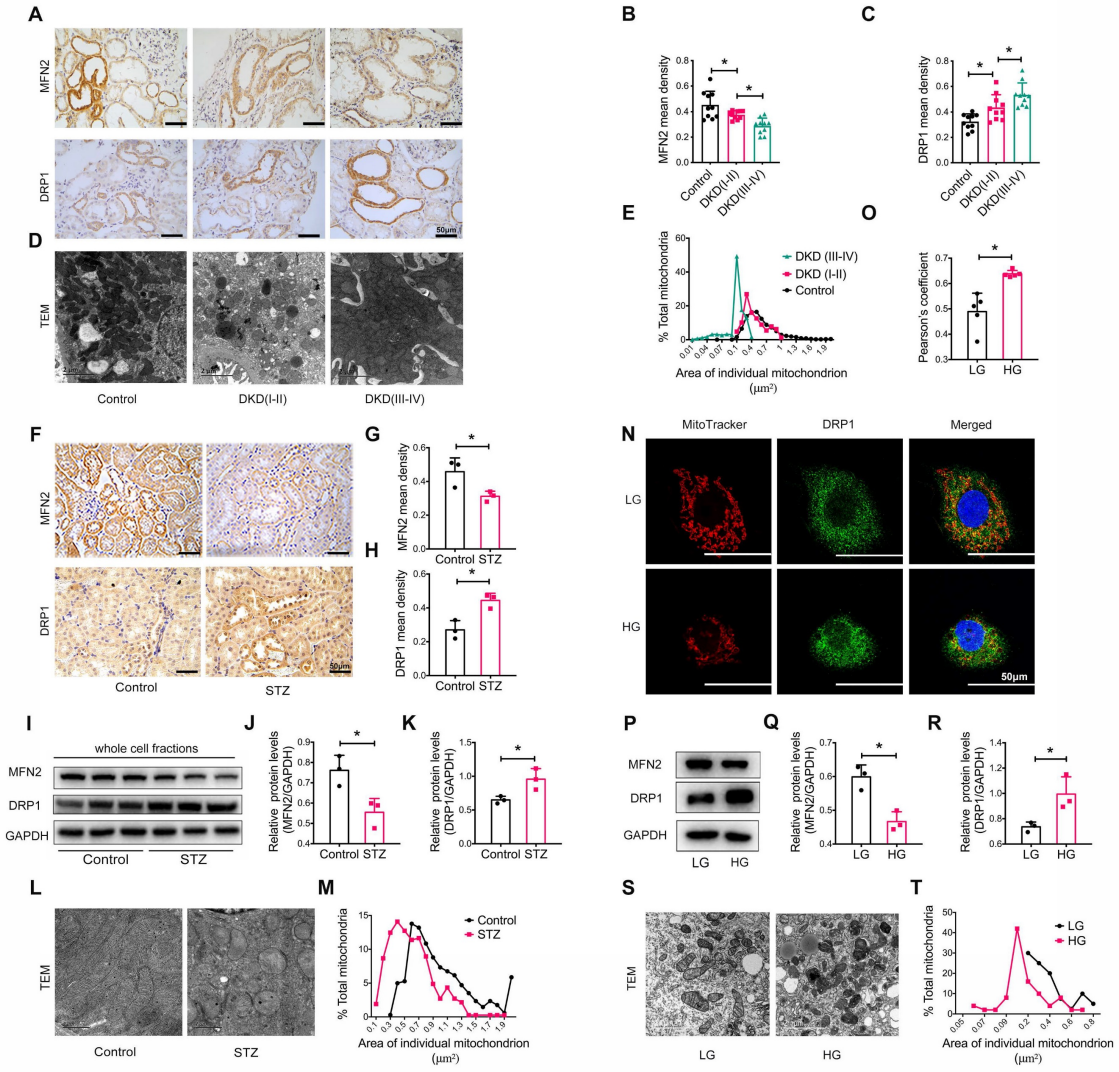
** Tubular mitochondrial dysfunction in patients with DKD, diabetic mice, and high glucose stimulated HK-2 cells.** (A) Immunohistochemistry analysis of tubular MFN2 and DRP1 in healthy controls and patients with DKD (I-II) and DKD (III-IV) (Scale bar, 50μm). (B,C) Quantification of MFN2 and DRP1 in different groups, n=10. (D) TEM analysis of mitochondrial morphology in healthy controls and patients with DKD (I-II) and DKD (III-IV) (Scale bar, 2μm). (E) Changes in the mitochondrial cross-section area in different groups. At least 2000 mitochondria in each group were analyzed. (F) Immunohistochemistry analysis of tubular MFN2 and DRP1 in STZ-induced diabetic mice (Scale bar, 50μm). (G,H) Quantification of MFN2 and DRP1, n=3. (I) Western blot analysis of MFN2 and DRP1 in renal cortices of controls and STZ-induced diabetic mice. (J,K) Quantification of MFN2 and DRP1, n=3. (L) TEM analysis of mitochondrial morphology (Scale bar, 1μm). (M) Changes in the mitochondrial cross-section area. At least 300 mitochondria in each group were analyzed. (N) Immunofluorescence of MitoTracker and DRP1 in low or high glucose stimulated HK-2 cells (Scale bar, 50μm). (O) Pearson's correlation coefficient of MitoTracker and DRP1 of different groups, n=5. (P) Western blots of MFN2 and DRP1 in low or high glucose stimulated HK-2 cells. (Q,R) Quantification of MFN2 and DPR1, n=3. (S) TEM analysis of mitochondrial morphology in low or high glucose stimulated HK-2 cells (Scale bar, 2μm). (T) Changes in the mitochondrial cross-section area. At least 40 mitochondria in each group were analyzed. Data are the mean ± SD, * *P* < 0.05.

We also assessed the size and morphology of mitochondria in the renal tubules. Immunohistochemistry revealed the decreased levels of the mitochondrial fusion-related protein mitofusion 2 (MFN2) and the increased levels of the dynamin-related protein-1 (DRP1) (**Figure 2A-C**). TEM showed marked mitochondrial fragmentation in patients with DKD compared to that in healthy controls (**Figure 2D-E**). Similar observations were made in the kidneys of STZ-induced diabetic mice with DKD (**Figure 2F-M**). In vitro, the addition of high glucose to HK-2 cells induced the translocation of DRP1 to the mitochondria (**Figure 2N-O**) and an imbalance in MFN2 and DRP1 levels (**Figure 2P-R**), consistent with mitochondrial fragmentation (**Figure 2S and T**). Taken together, these findings reveal that a disruption of MAM integrity and mitochondrial dysfunction are closely related to the development of tubular injury in DKD.

### Identification of the key genes associated with tubular injury in DKD

To investigate the possible molecular mechanism of tubular injury under diabetic conditions, we analyzed the GSE30529 dataset from the GEO database, which contains tubular tissues from 10 DKD and 12 normal control samples, and performed differentially expressed genes (DEGs) analysis with 3378 DEGs, including 2165 upregulated and 1213 downregulated genes in DKD compared with the control (**Figure S4A**). We performed KEGG pathway enrichment analyses of the DEGs to better understand their biological functions. The top ten most enriched and statistically significant KEGG pathways were identified (**Figure S4B**). The most gene-enriched pathways were “Pathways in cancer” (115 genes, *P* = 8.33e-07) and “PI3K-Akt signaling pathway” (99 genes, *P* = 1.35e-05). As prior studies have showed that the PI3K/AKT signaling pathway play a role in cell proliferation, differentiation, and glucose metabolism, and is responsible for obesity 42, diabetes 43, and DKD 44, 45, we extracted and imported the gene cluster of the “PI3K/AKT signaling pathway” KEGG term into the Cytoscape for further analysis. We calculated the 'betweenness' value of each gene in the gene cluster (which is a measure of centrality within a network) and identified *Mapk1* as a potentially critical key node in the development of tubular injury in DKD (**Figure S4C**).

**Figure 3:**
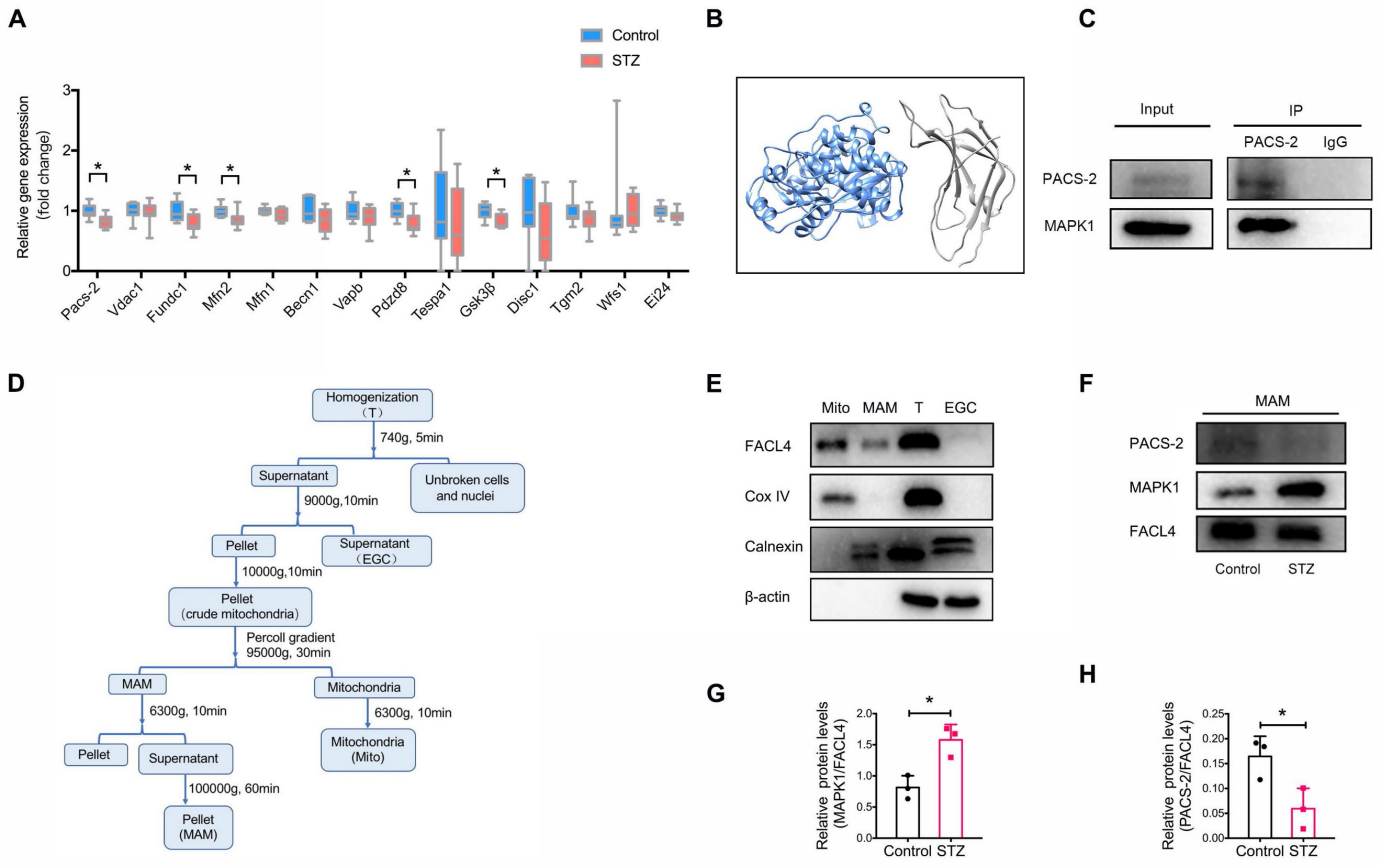
** Identification of potential genes associated with the disruption of MAM in diabetic mice.** (A) Relative expression of genes encoding MAM-related proteins in the kidney cortex of diabetic mice and normal controls by RNA sequencing, n=9-10. (B) Three-dimensional structures showing that MAPK1 and PACS-2 likely form a protein complex, as predicted by Uni-Fold. MAPK1, in blue, is predicted to interact with the N terminus domain of PACS-2 formed between residues 1 and 154, which are colored in grey. (C) HK-2 cells stably expressing MAPK1 were immunoprecipitated using protein A/G beads. The left panels show immunoblots of MAPK1 and PACS-2 as input materials. The two panels on the right show the combination of PACS-2 and MAPK1 detected by immunoblotting following immunoprecipitation. (D) A diagram of isolation of MAM from the mouse kidney cortex. (E) Western blot analysis of MAM markers including FACL4, Cox-IV, and Calnexin. T, total cell lysate; EGC: ER, Golgi, Cytoplasm; Mito, mitochondria. (F-H) Western blot analysis of MAPK1 and PACS-2 in MAM isolated from control and diabetic mice (n = 3). Data are the mean ± SD, * *P* < 0.05.

As the disruption of MAM is closely associated with the development of DKD, we next attempted to identify genes regulating MAM under high glucose conditions by performing transcriptome sequencing of the kidney cortex of diabetic mice and controls. Filtering for MAM-related proteins 15, 20, we found that the expression of *Pacs-2*, encoding phosphofurin acidic cluster sorting protein-2*,* was largely downregulated in diabetic kidneys compared to controls (**Figure 3A, Figure S5**), suggesting *Pacs-2* may be a critical gene mediating the disruption of MAM in DKD. To explore the possible interaction between MAPK1 and PACS-2, we first used Uni-Fold, a protein structure prediction tool to predict protein structure, and the three-dimensional structures showed that MAPK1/PACS-2 was likely to form protein complexes (**Figure 3B**). We verified the interaction between MAPK1 and PACS-2 under low glucose conditions using immunoprecipitation in HK-2 cells (**Figure 3C**).

Next, to clarify the cellular regional localization of MAPK1 and PACS-2, MAM was isolated from the kidney cortex of diabetic and control mice by Percoll density gradient fractionation (**Figure 3D**). MAMs were successfully purified based on Western blotting for the markers: Fatty Acid-CoA Ligase 4 (FACL4; MAM and mitochondria), Cytochrome c oxidase IV (Cox IV; mitochondria only), and Calnexin (endoplasmic reticulum and MAM; **Figure 3E**). MAPK1 and PACS-2 were found in MAM by Western blotting with MAPK1 levels being increased and PACS-2 levels being decreased in kidney cortices from diabetic animals compared to controls (**Figure 3F-H**).

**Figure 4:**
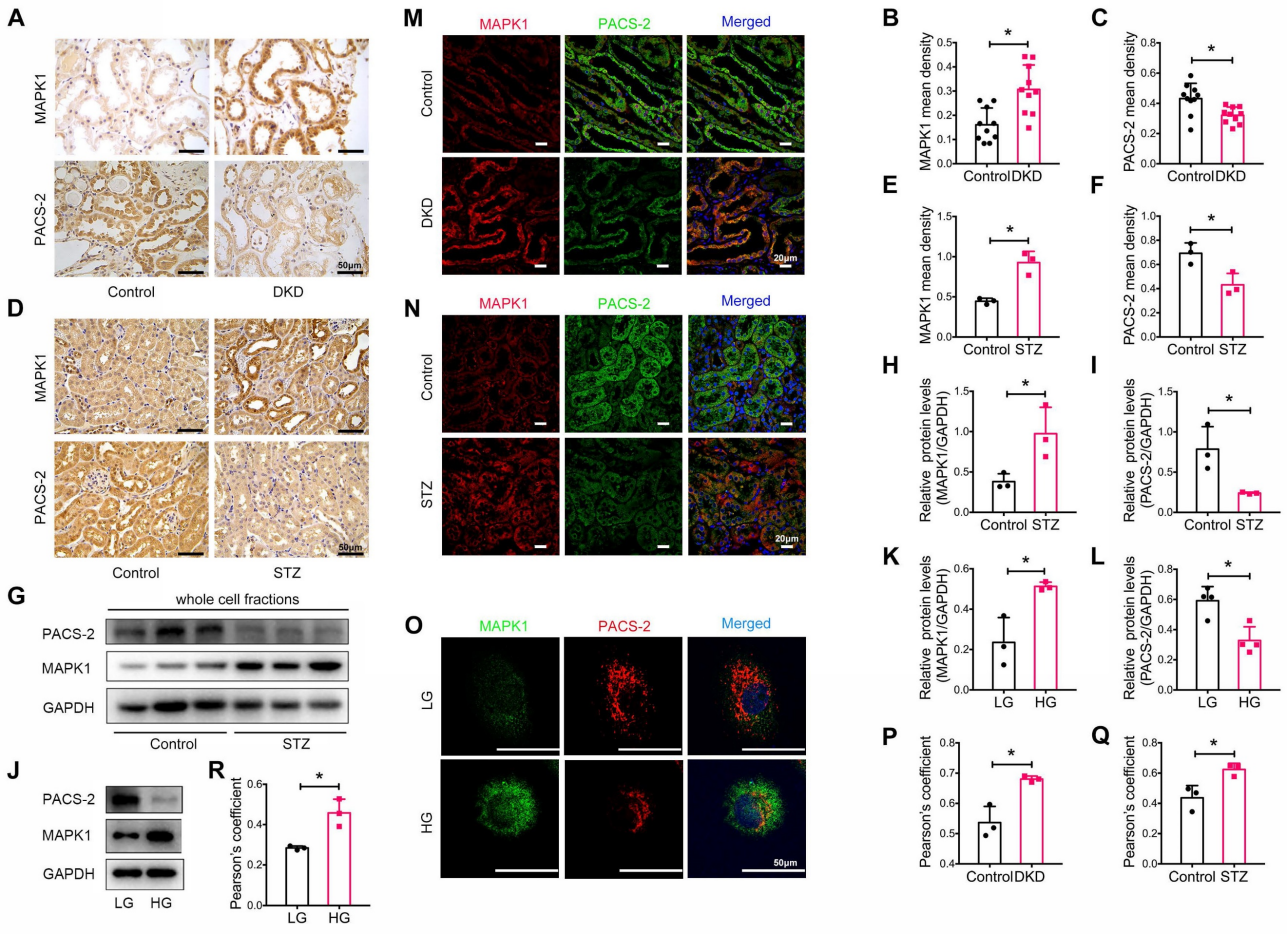
** Expression and co-localization of MAPK1 and PACS-2 in renal tubules of patients with DKD, diabetic mice, and high glucose stimulated HK-2 cells.** (A-C) MAPK1 and PACS-2 expression in patients with DKD by immunohistochemistry, n=10 (Scale bar, 50μm). (D-F) Immunohistochemical staining shows MAPK1 and PACS-2 expression in STZ-induced diabetic mice kidneys, n=3 (Scale bar, 50μm). (G-I) Western blot analysis detects the expression of MAPK1 and PACS-2 in the kidney cortex of control and diabetic mice, n=3. (J-L) Western blot analysis detects the expression of MAPK1 and PACS-2 in low or high glucose stimulated HK-2 cells, n=3-4. (M-O) Immunofluorescence detection of co-localization of MAPK1/PACS-2 in renal tubules in controls and patients with DKD (Scale bar, 20μm), in diabetic mice (Scale bar, 20μm), and low or high glucose stimulated HK-2 cells (Scale bar, 50μm). (P-R) Pearson's correlation coefficient of MAPK1/PACS-2 in controls and patients with DKD or diabetic mice and low or high glucose stimulated HK-2 cells, n=3. Data are the mean ± SD, * P < 0.05.

We also determined the expression levels of MAPK1 and PACS-2 in patients with DKD, whose clinical characteristics are presented in **Table S2**. By immunostaining of tissue sections, we found that MAPK1 was upregulated while PACS-2 was downregulated in the renal tubules of patients with DKD compared with that in healthy controls (**Figure 4A-C).** Correlation analysis showed that increased MAPK1 was negatively correlated with eGFR, whereas the levels of PACS-2 were positively correlated with eGFR (**Figure S6A-B**), suggesting that the upregulation of MAPK1 and down-regulate of PACS-2 may contribute to the impairment of renal function in patients with DKD. Similarly, in STZ-induced diabetic mouse kidneys and high glucose-stimulated HK-2 cells, IHC and Western blot analyses revealed an upregulation of MAPK1 and a downregulation of PACS-2 in renal tubules (**Figure 4D-L**). Furthermore, two-color immunofluorescence showed tight co-localization of MAPK1/PACS-2 in the tubular cells of diabetic patients and mice, as well as in HK-2 cells exposed to high glucose (**Figure 4M-R**). Taken together, these findings suggested that increased MAPK1 in DKD may contribute to the disruption of MAM by down-regulating PACS-2.

### MAPK1 mediates the disruption of MAM integrity and mitochondrial dysfunction in STZ-induced diabetic mice

**Figure 5:**
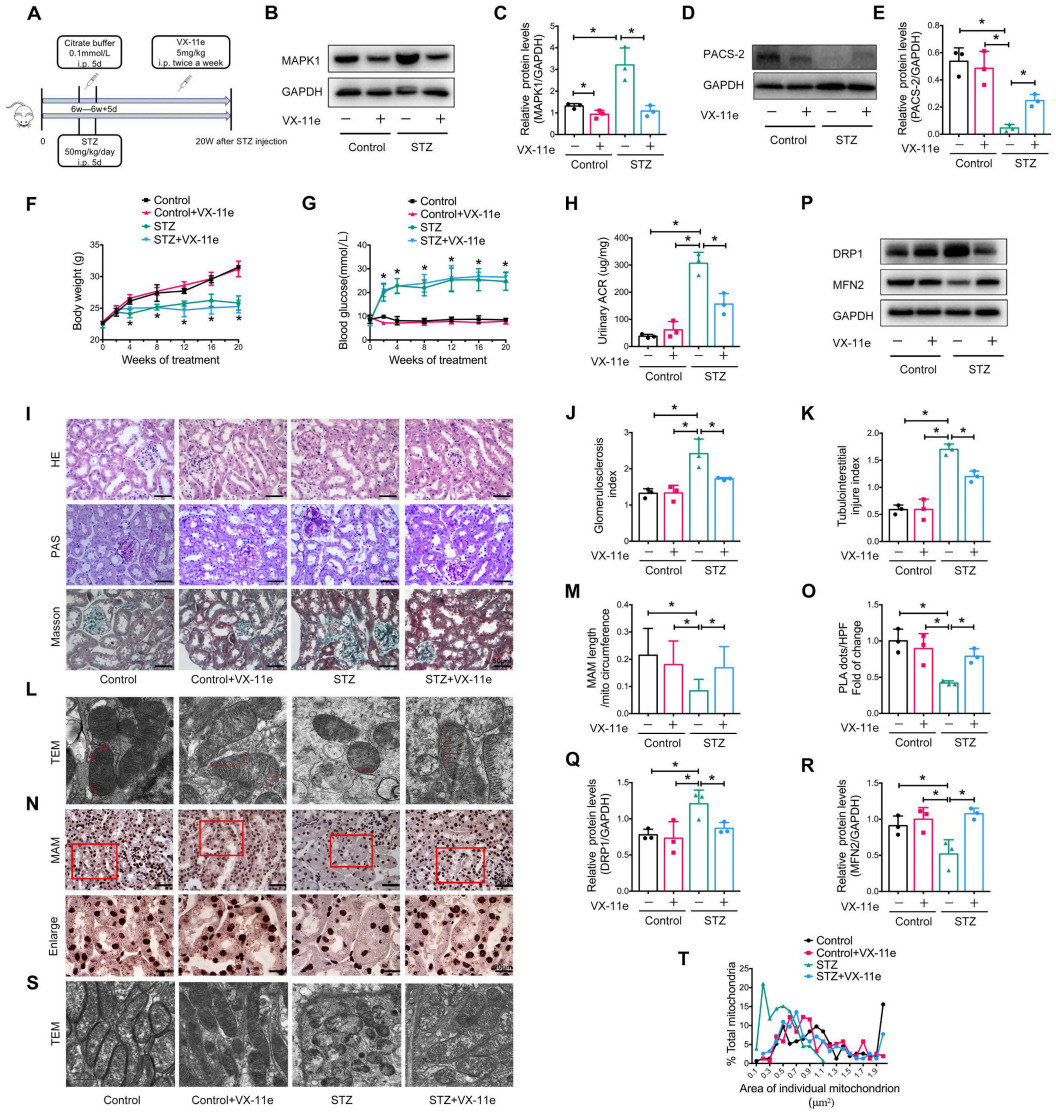
** Inhibition of MAPK1 increases PACS-2 and improves diabetic kidney injury in STZ-induced diabetic mice.** (A) A diagram of the mouse model. (B-E) Western blot analysis of the expression of MAPK1 and PACS-2 in the kidney cortex of mice in different groups. (F-H) Effect of inhibiting MAPK1 on body weight, blood glucose, and urinary ACR of mice in different groups. (I) Effect of inhibiting MAPK1 on pathological changes determined by H&E, PAS, and Masson staining (scale bar, 50 μm). (J-K) Effect of inhibiting MAPK1 on glomerulosclerosis index (GSI) and tubulointerstitial injury index (TII). (L) TEM analysis of the length of MAM in the renal tubules of mice in different groups (Scale bar, 0.5μm). (M) Quantification of MAM length. 45 MAM in each group were analyzed. (N) In-situ PLA detection of the number of MAM in the tubular cells of mice in different groups (Scale bar, 50/20μm). (O) Quantitative analysis of PLA dots/ HPF in mice of different groups, n=3. (P-R) Western blot analysis of DRP1, MFN2 expression in renal cortices of mice in different groups. (S) TEM analysis of mitochondrial morphology in renal tubules of mice in different groups (Scale bar, 1μm). (T) The mitochondrial cross-section area. At least 150 mitochondria in each group were analyzed. Data are the mean ± SD, n=3, * *P* < 0.05.

To further explore whether MAPK1 mediates the disruption of MAM integrity and mitochondrial dysfunction under diabetic conditions, we intraperitoneally injected mice with VX-11e, a MAPK1 inhibitor (**Figure 5A**). The results showed that treatment with VX-11e markedly inhibited the expression of MAPK1 while increasing PACS-2 in the kidneys of STZ-induced diabetic mice (**Figure 5B-E**). This was related to a significant reduction in the urine albumin/creatinine ratio (ACR) without altering body weight and blood glucose levels in the diabetic mice (**Figure 5F-H**). Histologically, MAPK1 inhibition significantly improved renal pathological injury in diabetic mice by inhibiting mesangial matrix expansion and tubulointerstitial injury, as demonstrated by H&E, PAS, and Masson staining (**Figure 5I-K**).

Next, we examined the regulatory role of MAPK1 in the integrity of MAM and mitochondrial dysfunction in the kidneys of diabetic mice. TEM and PLA showed that blockade of MAPK1 significantly inhibited the disruption of MAM (**Figure 5L-O**). Western blot analysis also showed that treatment with VX-11e increased MFN2 expression but decreased DRP1 expression in diabetic kidneys (**Figure 5P-R**), thereby improving mitochondrial fragmentation (**Figure 5S-T**).

### MAPK1 mediates the disruption of MAM integrity and mitochondrial dysfunction in HK-2 cells exposed to high glucose

**Figure 6:**
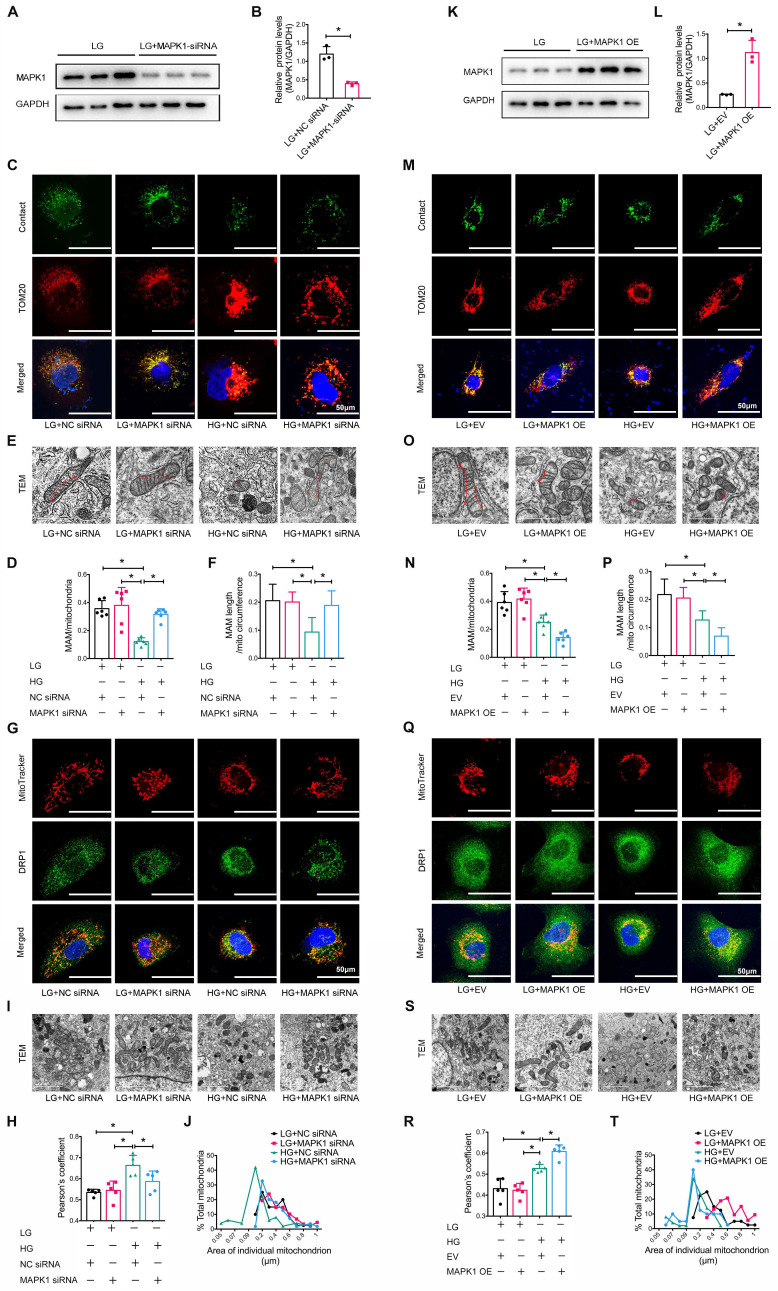
** Silencing or overexpression of* MAPK1* improves or aggravates MAM integrity and mitochondrial dysfunction in high glucose stimulated HK-2 cells.** (A-B) Western blot analysis of the expression of MAPK1 in HK-2 cells with or without *MAPK1* silencing, n=3. (C-D) Immunostaining of the MAM reporter (green) and TOM20 (red) in HK-2 cells (Scale bar, 50μm), n=6. (E-F) TEM analysis of the length of MAM in HK-2 cells, at least 10 MAM in each group were analyzed (Scale bar, 1μm). (G-H) Two-color immunofluorescence detects MitoTracker (red) and DRP1 (green) in HK-2 cells (Scale bar, 50μm), n=5. (I-J) TEM analysis of mitochondrial morphology in HK-2 cells (Scale bar, 2μm), at least 40 mitochondria in each group were analyzed. (K-L) Western blot analysis of the expression of MAPK1 in HK-2 cells with or without *MAPK1* overexpression, n=3. (M-N) Immunostaining of the MAM reporter (green) and TOM20 (red) in HK-2 cells (Scale bar, 50μm), n=6. (O-P) TEM analysis of the length of MAM in HK-2 cells, at least 10 MAM in each group were analyzed (Scale bar, 1μm). (Q-R) Two-color immunofluorescence of MitoTracker (red) and DRP1 (green) in HK-2 cells (Scale bar, 50μm), n=5. (S-T) TEM analysis of mitochondrial morphology in HK-2 cells (Scale bar, 2μm), at least 40 mitochondria in each group were analyzed. Data are the mean ± SD, * *P* < 0.05.

We also explored the regulatory role of MAPK1 in maintaining the integrity of MAM and mitochondrial dysfunction in HK-2 cells by knocking down *MAPK1* using siRNA (**Figure 6A-B**). Confocal microscopy clearly detected that silencing *MAPK1* blocked the HG-induced loss of colocalization of MitoTracker and ER markers in HK-2 cells (**Figure S7A-B**). More specifically, the use of a split-GFP-based gene-encoded fluorescent reporter also revealed that *MAPK1* knockdown significantly blocked the high glucose-induced disruption of MAM (**Figure 6C-D**). This was further confirmed by TEM (**Figure 6E-F**). We also found silencing* MAPK1* significantly inhibited the high glucose-induced translocation of DRP1 to the mitochondria (**Figure 6G-H**), thereby inhibiting mitochondrial fragmentation in HK-2 cells (**Figure 6I-J**).

To further delineate the regulatory role of MAPK1 in the integrity of MAM and mitochondrial dysfunction, we constructed stable *MAPK1*-overexpressing HK-2 cells (**Figure 6K-L**) and found that overexpression of *MAPK1* largely promoted HG-induced inhibition of the co-localization of MitoTracker and ER markers in HK-2 cells (**Figure S7C-D**). The split-GFP-based gene-encoded fluorescent reporter also revealed that overexpression of* MAPK1* significantly aggravated high glucose-induced disruption of MAM by largely reducing MAM content (**Figure 6M-N**). Again, TEM confirmed that the overexpression of* MAPK1* further aggravated the disruption of MAM in HK-2 cells exposed to high glucose (**Figure 6O-P**). *MAPK1* overexpression also worsened mitochondrial function in HG-stimulated HK-2 cells (**Figure 6Q-T**). These results suggest that MAPK1 mediates MAM disruption and mitochondrial dysfunction under high glucose conditions.

### MAPK1 induces the disruption of MAM integrity and mitochondrial dysfunction in HK-2 cells via a PACS-2-dependent mechanism

**Figure 7:**
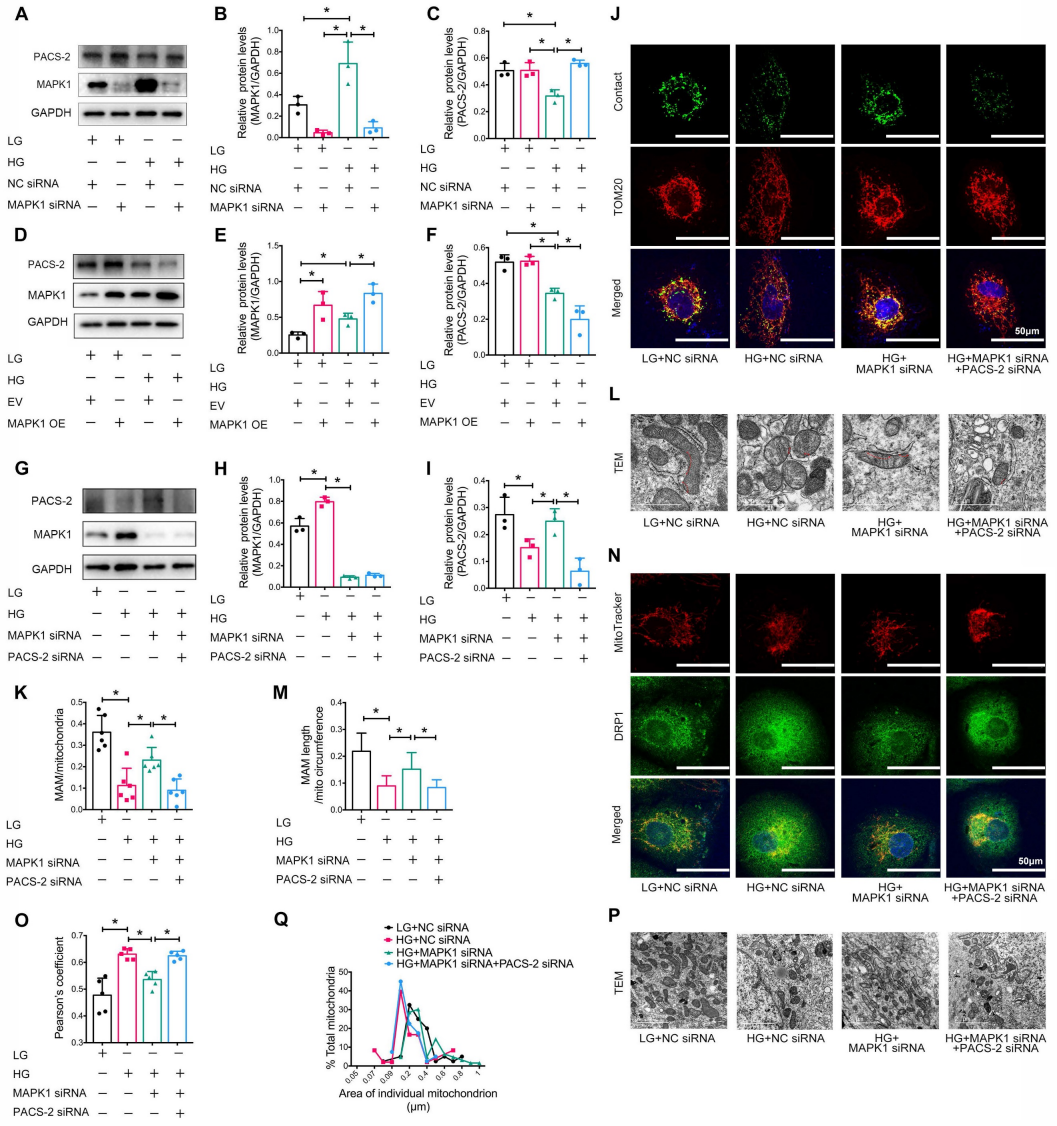
** Silencing* PACS-2* reverses the protective effect of* siMAPK1* on MAM disruption and mitochondrial dysfunction in high glucose stimulated HK-2 cells.** (A-C) Western blot analysis of the expression of MAPK1 and PACS-2 in HK-2 cells treated with or without *siMAPK1*, n=3. (D-F) Western blot analysis of MAPK1 and PACS-2 expression in HK-2 cells with or without overexpressing *MAPK1*, n=3. (G-I) Western blot analysis of the expression of MAPK1 and PACS-2 in HK-2 cells treated with or without *siMAPK1*+*siPACS-*2, n=3. (J-K) Immunostaining of the MAM reporter (green) and TOM20 (red) in HK-2 cells (Scale bar, 50μm), n=6. (L-M) TEM analysis of the length of MAM in HK-2 cells, at least 10 MAM in each group were analyzed (Scale bar, 1μm). (N-O) Two-color immunofluorescence of MitoTracker (red) and DRP1 (green) in HK-2 cells (Scale bar, 50μm), n=5. (P-Q) TEM analysis of mitochondrial morphology in HK-2 cells (Scale bar, 2μm), at least 40 mitochondria in each group were analyzed. Data are the mean ± SD; * P < 0.05.

Next, we examined the downstream mechanism of MAPK1-mediated disruption of MAM integrity and mitochondrial dysfunction in HK-2 cells. As shown in **Figure 7A-F**, knockdown of *MAPK1* prevented the high glucose-induced loss of PACS-2, which was reversed by overexpression of *MAPK1*. To further confirm the regulatory role of MAPK1 in MAM integrity and mitochondrial dysfunction via a PACS-2-dependent mechanism, we constructed *MAPK1* and* PACS-2* double knockdown HK-2 cell lines, as shown in **Figure 7G-I**. Interestingly, in the double knockdown of *MAPK1* and* PACS-2* HK-2 cells, silencing *PACS-2* resulted in the loss of the protective effect of knockdown of *MAPK1* on high glucose-induced disruption of MAM, as determined by confocal microscopy (**Figure S7E-F**), the split-GFP-based gene-encoded fluorescent reporter (**Figure 7J-K**) and TEM (**Figure 7L-M**). Further studies showed that double knockdown of *MAPK1* and* PACS-2* reversed the protective effect of *MAPK1* knockdown against HG-induced DRP1 recruitment and mitochondrial fragmentation in HK-2 cells (**Figure 7N-Q**). These results revealed a necessary role for the MAPK1-PACS-2 regulatory axis in maintaining MAM integrity and mitochondrial dysfunction under high glucose conditions.

**Figure 8:**
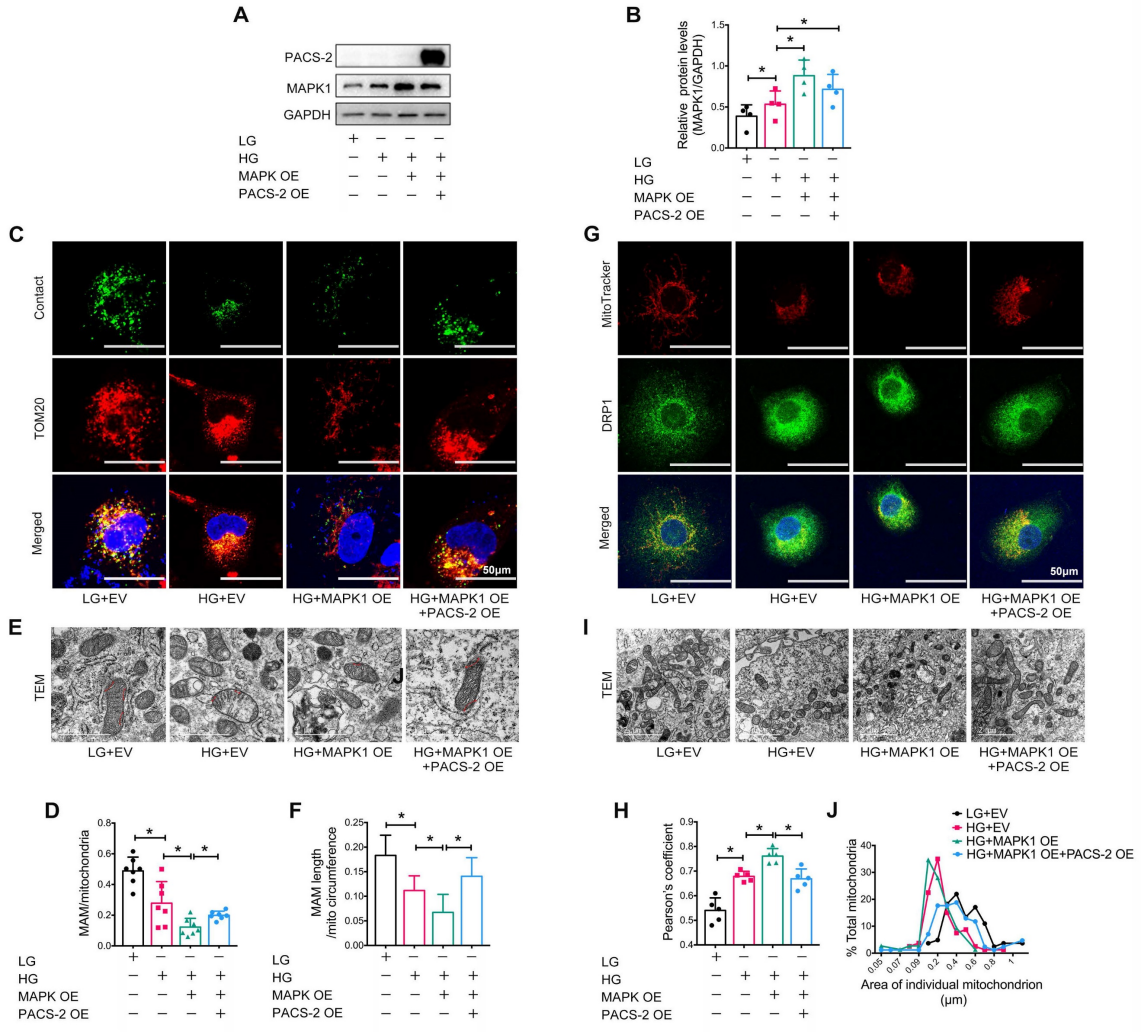
***PACS-2* overexpression protects against MAM disruption and mitochondrial dysfunction induced by overexpressing *MAPK1* in high glucose stimulated HK-2 cells.** (A-B) Western blot analysis of the expression of MAPK1 and PACS-2 in HK-2 cells with or without double overexpressing *MAPK1* and *PACS-2*, n=4. (C-D) Immunostaining of the MAM reporter (green) and TOM20 (red) in HK-2 cells (Scale bar, 50μm), n=7. (E-F) TEM analysis of the length of the MAM in HK-2 cells, at least 10 MAM in each group were analyzed (Scale bar, 1μm). (G-H) Two-color immunofluorescence analysis of MitoTracker (red) and DRP1 (green) in HK-2 cells (Scale bar, 50μm), n=5. (I-J) TEM analysis of mitochondrial morphology in HK-2 cells (Scale bar, 2μm), at least 40 mitochondria in each group were analyzed. Data are the mean ± SD; * *P* < 0.05.

To further confirm the regulatory role of MAPK1-PACS-2 in MAM integrity and mitochondrial dysfunction, we generated *MAPK1* and* PACS-2* double-overexpression HK-2 cells (**Figure 8A-B**). In contrast to the double knockdown cells, confocal microscopy, reporter assays, and TEM revealed that HK-2 cells overexpressing *MAPK1* developed significant MAM disruption, DRP1 recruitment, and mitochondrial fragmentation under high glucose conditions, which were reversed by overexpressing* PACS-2* (**Figure S7G-H, Figure 8**). These results demonstrated that MAPK1 mediates the disruption of MAM integrity and mitochondrial dysfunction in HK-2 cells under high glucose conditions by downregulating PACS-2.

## Discussion

Here, we report that the development of DKD is linked to a disruption in MAM, in agreement with previous studies 25, 46. Importantly, we also found that MAM disruption in the diabetic kidney was tightly associated with marked upregulation of MAPK1 and downregulation of PACS-2, providing a link between MAPK1 and PACS-2 in the disruption of MAM under diabetic conditions. Functional studies revealed that the blockade of MAPK1 upregulated PACS-2, thus inhibiting the disruption of MAM integrity and improving renal injury in diabetic mice by significantly inhibiting proteinuria and pathological renal damage. These findings reveal a key role of the MAPK1-PACS-2 axis in regulating the disruption of MAM during the pathogenesis of DKD.

PACS-2 is a key regulator of MAM 47, 48, where it plays a physical role in tethering mitochondria to the ER, and has also been implicated in metabolic diseases like obesity and insulin resistance 18, 49. Consistent with a recent report 25, we found that the loss of PACS-2 in the renal tubules of patients with DKD and STZ-induced diabetic mice was associated with the disruption of MAM and mitochondrial dysfunction. Functionally, overexpression of *PACS-2* protected against high glucose-induced disruption of MAM, which was reversed by knocking down *PACS-2*, revealing a protective role of PACS-2 in the pathogenesis of DKD. This is also consistent with a recent finding that the *Pacs-2* knockout mice develop more severe DKD associated with the disruption of MAM, mitochondrial dysfunction, renal apoptosis, and fibrosis, which is reversed by overexpressing *Pacs-2* 50.

A significant finding of this study was the identification of the negative regulatory role of MAPK1 in PACS-2 expression during the development of DKD. We found that MAPK1 and PACS-2 were likely to form protein complexes, as predicted by Uni-Fold, and confirmed their interactions by immunoprecipitation. More importantly, in vitro and in vivo experiments showed that the loss of PACS-2 was associated with the upregulation of MAPK1 in diabetic mouse kidneys and HK-2 cells exposed to high glucose. We also found that under diabetic conditions, the disruption of MAM was mediated by MAPK1 via a PACS-2-dependent mechanism. This was supported by the finding that knockdown of *MAPK1* increased PACS-2 and inhibited high glucose-induced disruption of MAM integrity in HK-2 cells, which was blocked by silencing *PACS-2*. In contrast, MAPK1 downregulated PACS-2 while promoting high glucose-induced disruption of MAM integrity in HK-2 cells, which was reversed by overexpressing *PACS-2*. Thus, MAPK1 negatively regulates the expression of PACS-2, resulting in the disruption of MAM integrity under diabetic conditions.

We also found that MAPK1 regulated mitochondrial fragmentation, which was inhibited by overexpressing *PACS-2*, suggesting that MAPK1-mediated mitochondrial fragmentation is PACS-2-dependent. Prior work has shown that mitochondrial fragmentation in *PACS-2*-depleted cells occurs via a caspase-generated fragment of BAP31, which promotes mitochondria fission by regulating DRP1 23. Furthermore, it has been reported that glucose reduces MAM, subsequent mitochondrial fission, and restoration of MAM reduction reverses glucose-induced mitochondrial fragmentation in the liver 51. In line with these conclusions, our results suggest that glucose upregulates MAPK1, inducing mitochondrial fragmentation by inhibiting PACS-2 expression, enhancing DRP1 recruitment to the mitochondria and inducing MAM disruption.

MAPK1 is mainly present in the cytoplasm and nucleus and has been found to be present in the mitochondria of heart, brain, and kidney cells 52-54. In this study, we identified the localization of MAPK1 in MAM. Under diabetic conditions, MAPK1 in MAM was activated, which caused the disruption of MAM integrity and mitochondrial dysfunction by downregulating the expression of PACS-2, suggesting functional MAPK1/PACS-2 signaling at MAM.

Current findings on the changes in MAM integrity caused by high glucose are inconsistent. Related studies have reported that MAM integrity is disrupted in tubular cells under high glucose conditions and that disulphide-bond A oxidoreductase-like protein (DsbA-L) plays an anti-apoptotic role by maintaining the integrity of MAM 55. High glucose leads to increased MAM formation in tubular cells, and the reticulon 1A protein (RTN1A) aggravates tubular injury in DKD by enhancing MAM formation 56. The formation and content of MAM is highly plastic, depending on factors such as the metabolic status and stress conditions; therefore, inconsistent results have emerged from previous studies on MAM homeostasis in DKD, possibly due to differences in MAM detection methods, animal models, experimental parameters, and analytical methods. In this study, we used the split-GFP reporter gene to label MAM to achieve accurate observation of the MAM, which was confirmed to be reliable using TEM and confocal microscopy. Therefore, the use of a split-GFP reporter may be a better method for detecting MAM.

## Conclusions

DKD is associated with MAM disruption and mitochondrial dysfunction, which is mediated by MAPK1 via a PACS-2-dependent mechanism.

## Supplementary Material

Supplementary figures and tables.

## Abbreviations

DKD: Diabetic kidney disease
MAM: mitochondria-associated endoplasmic reticulum membrane
GEO: gene expression omnibus
ESRD: end-stage renal disease
ER: endoplasmic reticulum
PACS-2: phosphofurin acidic cluster sorting protein 2
MAPK1: mitogen-activated protein kinase 1
STZ: streptozotocin
i.p.: intraperitoneal injection
ATCC: american type culture collection
EGF: epidermal growth factor
LG: low glucose
HG: high glucose
HE: hematoxylin-eosin
PAS: periodic acid schiff
IHC: immunohistochemistry
IF: immunofluorescence
TEM: transmission electron microscopy
PLA: proximity ligation assay
DRP1: dynamin-related protein-1
MFN2: mitofusion 2
DEGs: differentially expressed genes
FACL4: fatty acid-coa ligase 4
Cox IV: cytochrome c oxidase IV
ACR: albumin/creatinine ratio
DsbA-L: disulphide-bond A oxidoreductase-like protein
RTN1A: reticulon 1A protein
