# Supplementary material for: MAPK1 Mediates MAM Disruption and Mitochondrial Dysfunction in Diabetic Kidney Disease via the PACS-2-Dependent Mechanism
Source: Int J Biol Sci. 2024 Jan 1;20(2):569–84. doi: 10.7150/ijbs.89291 (PMC10758092; doi:10.7150/ijbs.89291)
Supplement: Supplementary file 1 — Supplementary figures and tables. [file ijbsv20p0569s1.pdf]

Supplemental tables:

Table S1: Baseline characteristics of control and diabetic kidney disease (DKD) groups for transmission electron microscopy analysis.

	Control N=20	DKD (I-II) N=20	DKD (III-IV) N=20
Age (years)	52.40±1.15	53.20±3.39	47.90±1.58
Male (%)	40	50	55
BMI (kg/m2)	24.61±0.49	25.06±0.89	25.49±0.76
FBS (mmol/L)	5.19±0.15	6.29(4.79)	7.15±0.65
Systolic blood pressure (mm Hg)	122.50±2.77	146.10±4.27*	143.15±4.84*
Diastolic blood pressure (mm Hg)	76.50±1.51	76.80±2.90	84.95±2.74
Hemoglobin (g/L)	137.60±3.28	104.70±5.03*	107.15±4.67*
Cholesterol (mmol/L)	4.46±0.19	4.93±0.31	5.41±0.54
Albumin (g/L)	44.19±0.78	33.73±1.17*	29.87±1.87*
Serum creatinine (mg/dL)	65.50(8.98)	101.00(80.50)*	103.50(80.00)*
eGFR (MDRD) (ml/min/1.73 m^2)	97.42(8.98)	62.54±6.32*	63.00±6.00*
Protein/creatinine (urine, g/g)	0.07 (0.07)	3.68±0.37*	5.46(2.63)*

Data were presented as mean ± SD for normally distributed and as median (interquartile ranges) for non-normally distributed data. * $P < 0.05$ compared with Control; BMI, body mass index (kg/m2); FBS, fasting blood sugar (mmol/L).

Table S2: Baseline characteristics of control and DKD group for immunohistochemistry analysis.

	Control N=10	DKD N=10
Age (years)	52.70±1.30	53.90±5.02
Male (%)	70	60
BMI (kg/m2)	23.98±0.98	24.64 (7.07)
FBS (mmol/L)	4.98±0.19	6.33±0.69
Systolic blood pressure (mm Hg)	131.00(13.00)	135.40±5.69
Diastolic blood pressure (mm Hg)	78.50±3.57	76.27±3.53
Hemoglobin (g/L)	137.20±7.65	98.00 (36.00)*
Cholesterol (mmol/L)	4.49±0.24	5.38±0.62
Albumin (g/L)	43.79±0.91	30.55±2.15*
Serum creatinine (mg/dL)	69.30±4.12	269.60±36.77*
eGFR (MDRD) (ml/min/1.73 m^2)	99.88±2.37	25.83±4.99*
Protein/creatinine (urine, g/g)	0.07(0.09)	5.29±1.38*

Data were presented as mean ± SD for normally distributed and as median (interquartile ranges) for non-normally distributed data. * $P < 0.05$ compared with control.; BMI, body mass index (kg/m2); FBS, fasting blood sugar (mmol/L).

Supplemental Figures:

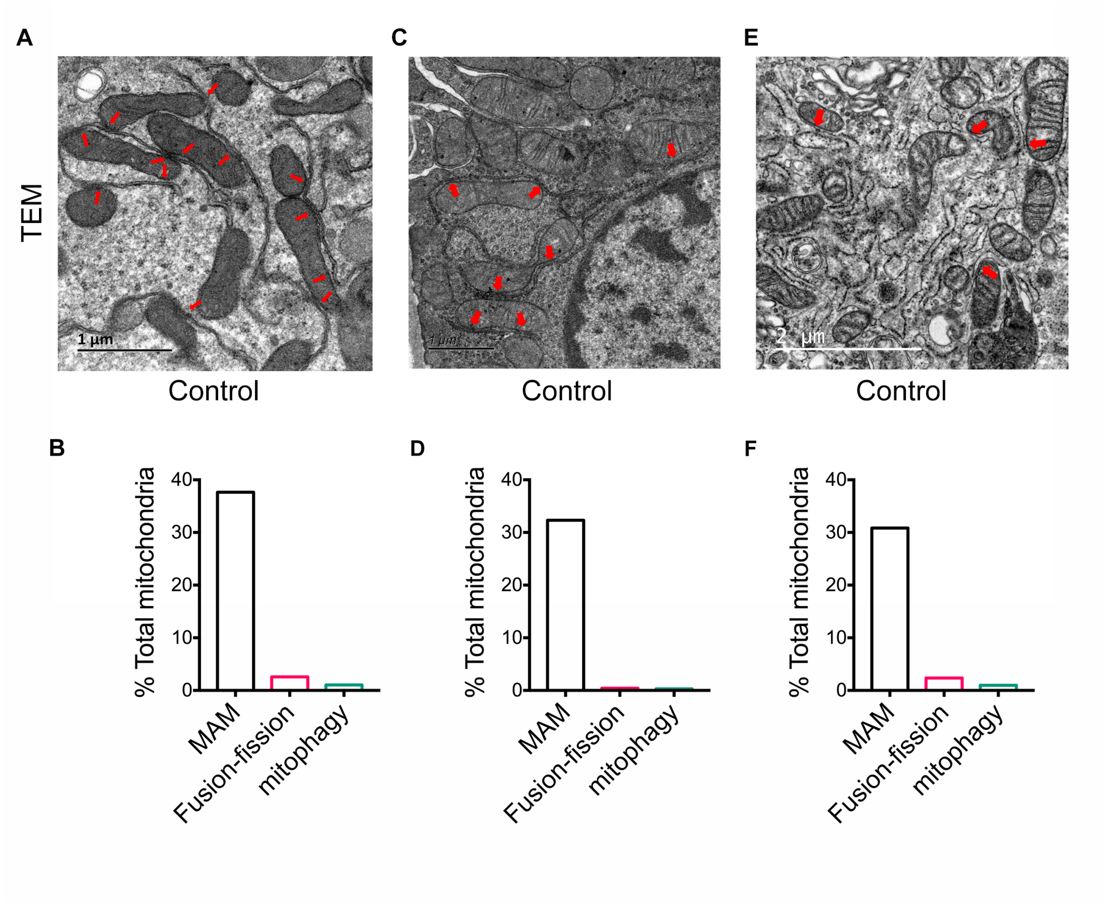

Figure S1. Statistics of mitochondrial morphology proportion.

(A) Mitochondrial morphology in renal tubules of healthy donors (Scale bar, 1 μm). (B) Percentage of different mitochondrial morphologies, at least 1000 mitochondria were analyzed. (C) Mitochondrial morphology in renal tubules of control mice (Scale bar, 1 μm). (D) Percentage of different mitochondrial morphologies, at least 700 mitochondria were analyzed. (E) Mitochondrial morphology in renal tubules of HK-2 cells exposed to low glucose (Scale bar, 2 μm). (F) Percentage of different mitochondrial morphologies, at least 500 mitochondria were analyzed.

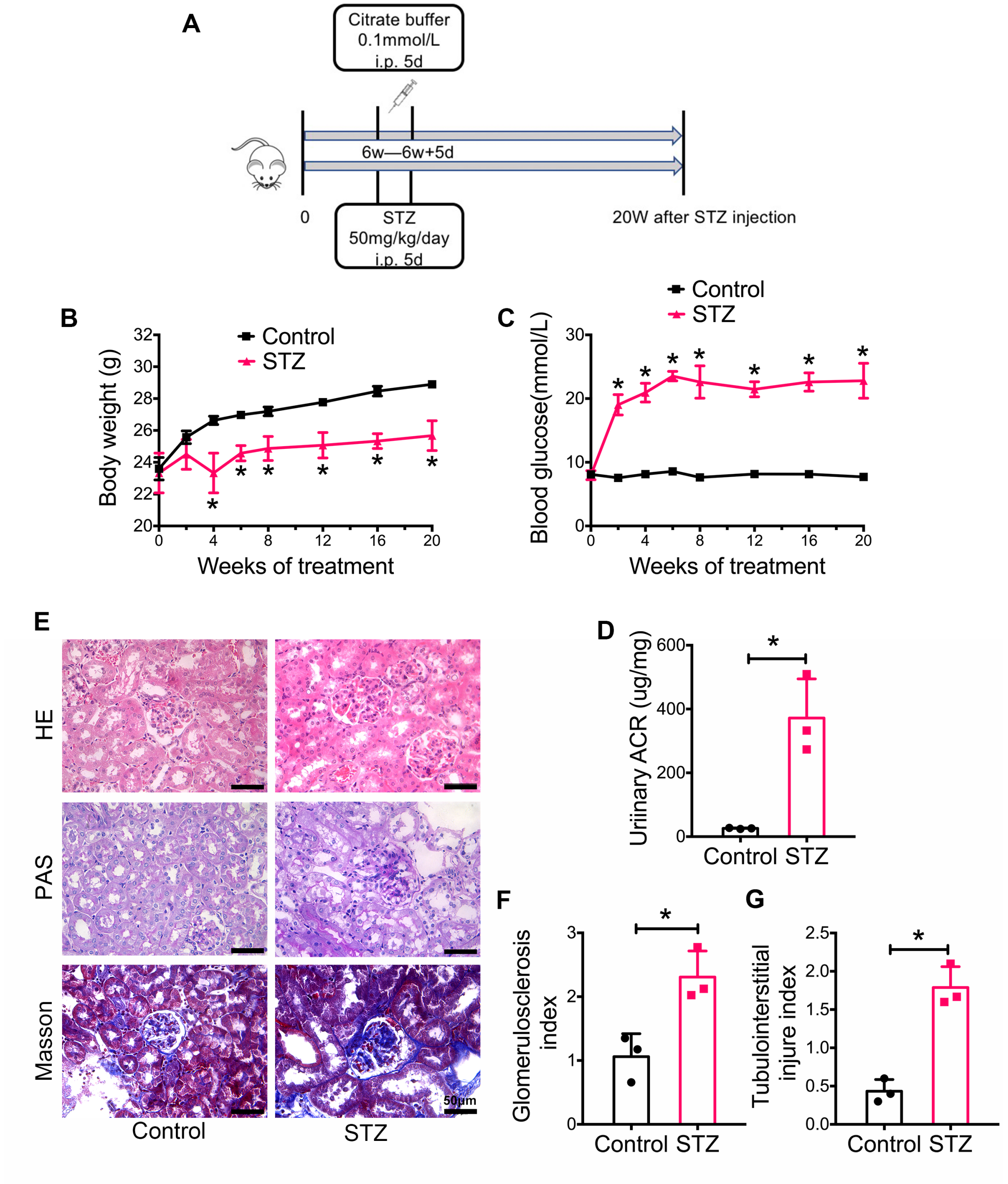

Figure S2. Biochemical and pathological changes in STZ-induced diabetic mice.

(A) A diagram of mouse model.(B) Body weight changes in control mice and STZ-induced diabetic mice. (C) Blood glucose levels. (D) Urinary ACR. (E) Pathological changes of kidney were showed by H&E, PAS and Masson staining (Scale bar, 50 μm).

(F) Glomerulosclerosis index (GSI). (G) Tubulointerstitial injure index (TII). The data are presented as the mean ± SD; n=3, * $P < 0.05$.

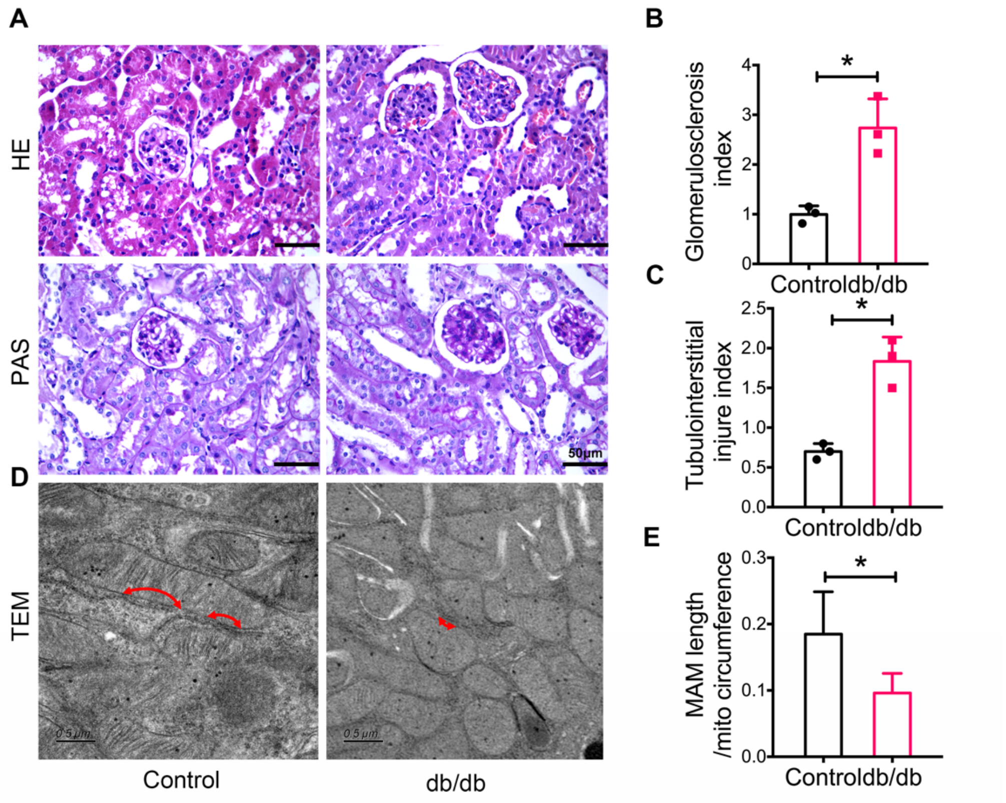

Figure S3. Pathological changes and MAM integrity in renal tubules of db/db mice. (A) Pathological changes of kidney were showed by H&E, PAS staining (Scale bar, 50μm). (B) Glomerulosclerosis index (GSI), n=3. (C) Tubulointerstitial injure index (TII), n=3. (D) TEM analysis of the length of MAM in renal tubules of db/db mice and controls. (E) The quantification of MAM length of db/db mice and controls, at least 24 MAM each group were analyzed. The data are presented as the mean ± SD; n=3, * $P < 0.05$.

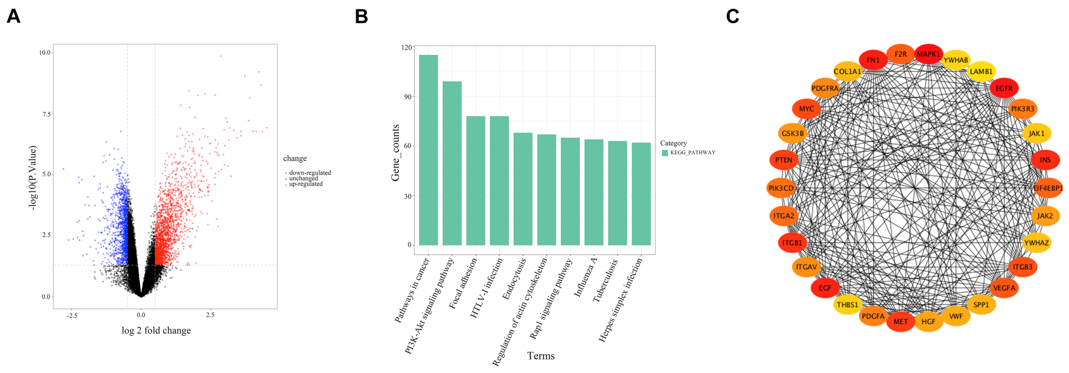

Figure S4. Identification of key gene related to DKD by bioinformatics analysis of GSE30529.

(A) Volcano plot of differentially expressed genes (DEGs) of GSE30529. (B) The top 10 most gene-enriched and statistically significant KEGG pathways. (C) Betweenness values in the gene cluster of "PI3K/AKT signaling pathway" KEGG term. Each node corresponds to a gene. Colors from yellow to red correspond to betweenness values from low to high.

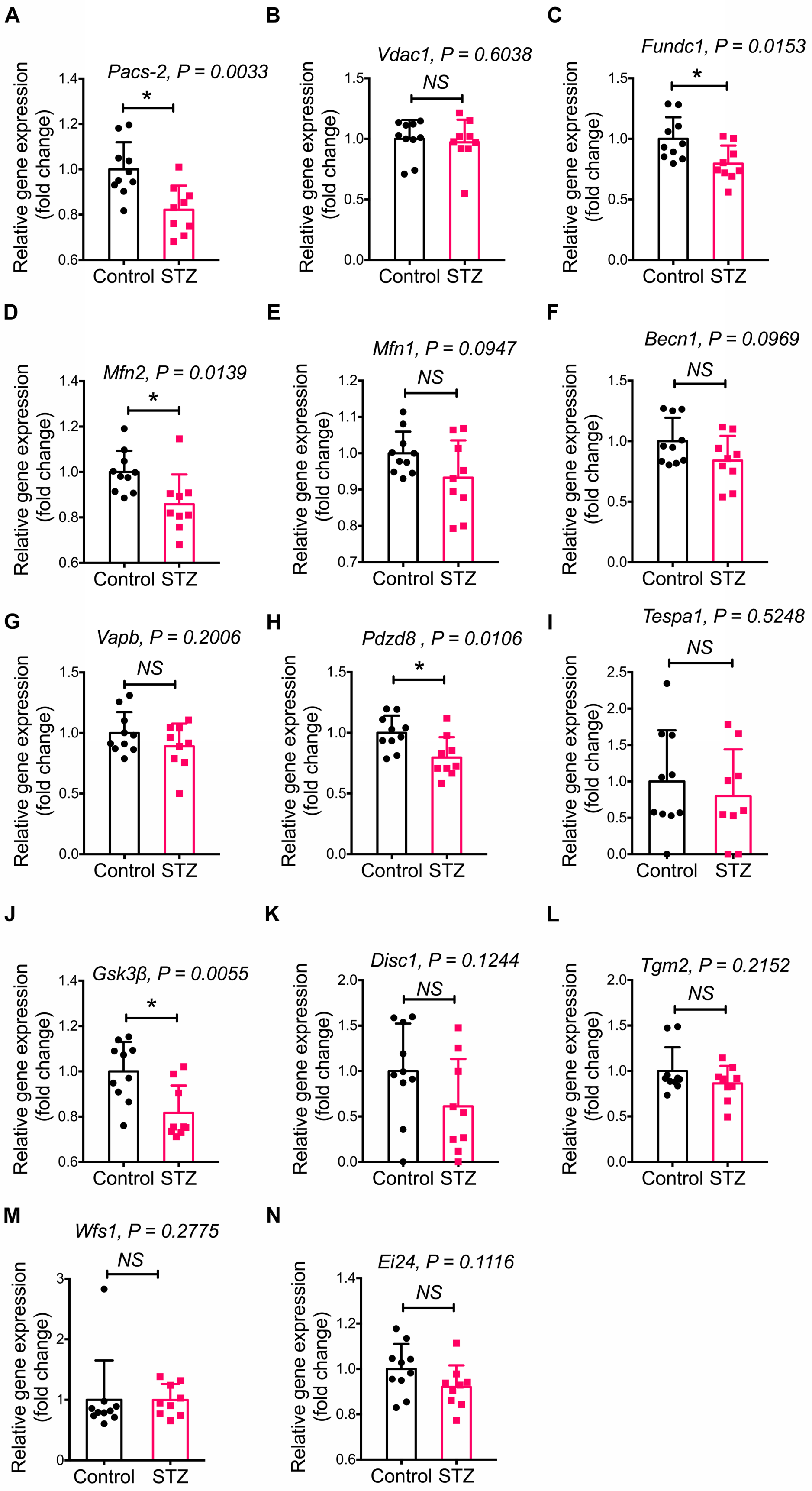
A
Pacs-2, P = 0.0033
B
Vdac1, P = 0.6038
C
Fundc1, P = 0.0153
D
Mfn2, P = 0.0139
E
Mfn1, P = 0.0947
F
Becn1, P = 0.0969
G
Vapb, P = 0.2006
H
Pdzd8 , P = 0.0106
I
Tespa1, P = 0.5248
J
Gsk3β, P = 0.0055
K
Disc1, P = 0.1244
L
Tgm2, P = 0.2152
M
Wfs1, P = 0.2775
N
Ei24, P = 0.1116
Relative gene expression (fold change)
Control
STZ
NS
*

Figure S5. The relative expression of genes encoding MAM-related proteins by RNA sequencing from renal cortex of diabetic mice and controls.

(A) *Pacs-2*. (B)*Vdac1*. (C) *Fundc1*.(D) *Mfn2*.(E) *Mfn1*. (F) *Becn1*. (G) *Vapb*. (H) *Pdzd8*. (I) *Tespa1*. (J) *Gsk3β*. (K) *Disc1*. (L) *Tgm2*. (M) *Wfs1*. (N) *Ei24*. The data are presented as the mean ± SD, n=9-10, * $P < 0.05$.

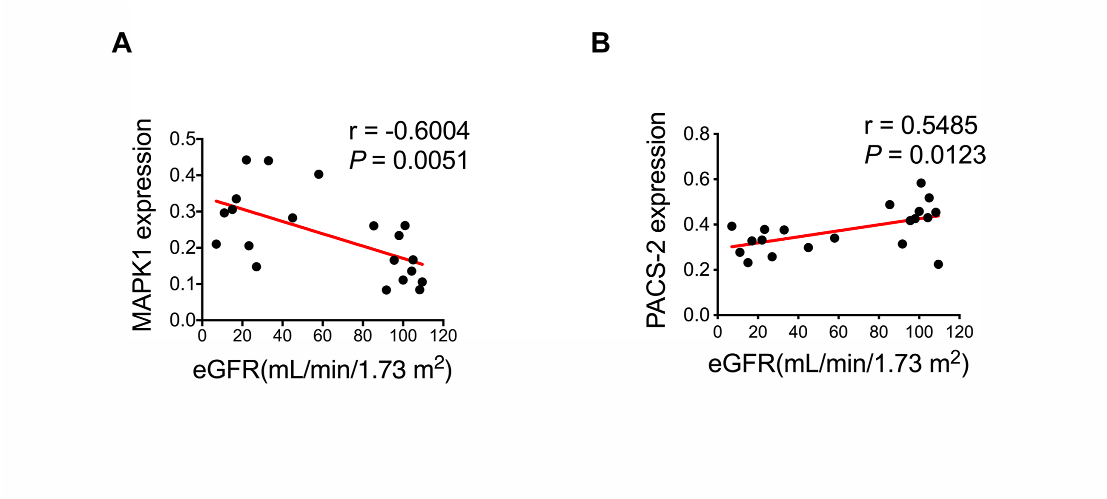

Figure S6. Correlation between the expression of MAPK1/PACS-2 by immunohistochemical quantitative analysis and renal function.

(A) Correlation analysis between MAPK1 expression and glomerular filtration rate. (B) Correlation analysis between PACS-2 expression and glomerular filtration rate.

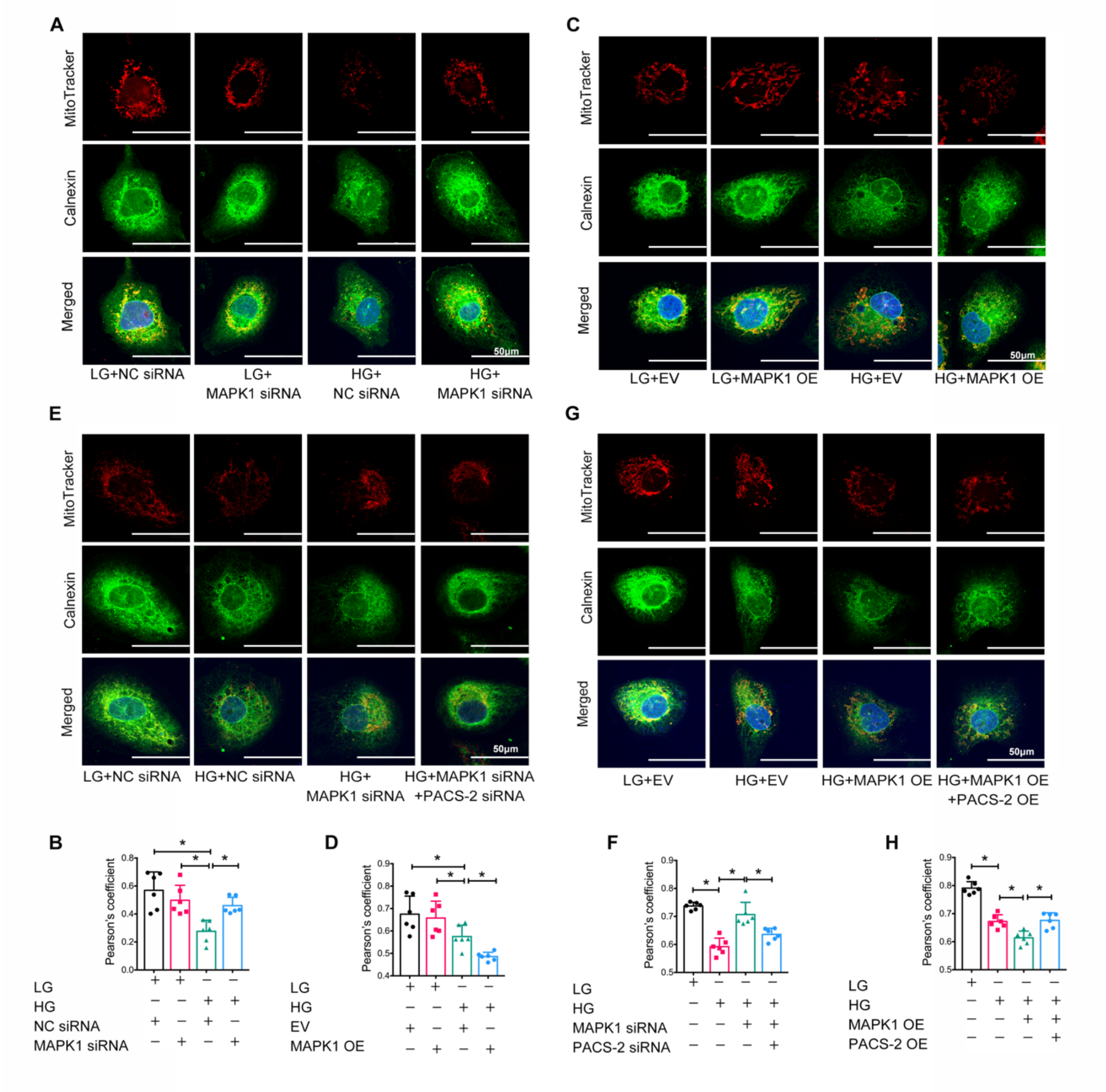

Figure S7. Two-color immunofluorescence co-localization of mitochondria and ER in HK-2 cells.

(A-B) Co-localization of mitochondria (MitoTracker, red) and ER (Calnexin, green) in HK-2 cells with or without *MAPK1* silencing (Scale bar, 50μm). (C-D) Co-localization of mitochondria and ER in HK-2 cells with or without *MAPK1* overexpression (Scale bar, 50μm). (E-F) Co-localization of mitochondria and ER in HK-2 cells with or without *siMAPK1* + *siPACS-2* (Scale bar, 50μm). (G-H) Co-localization of mitochondria and ER

in HK-2 cells with or without *MAPK1* and *PACS-2* double overexpressing (Scale bar, 50μm). The data are presented as the mean ± SD, n=6, * $P < 0.05$.

Supplemental Methods:

Analysis of GEO dataset

Firstly, we searched the Gene Expression Omnibus (GEO) database (https://www.ncbi.nlm.nih.gov/geo/) and obtained GSE30529 matrix format files, which contained 10 DKD and 12 normal tubular tissue samples. Then, we applied Differentially Expressed Genes (DEGs) analysis to compare the difference of the expression profiles between DKD and normal samples by GEO2R tool, a R-based web application that helps to analyze GEO data. The cut-off criteria for DEGs were set as $|\log FC| > 0.5$ and $P < 0.05$. The results were visualized with the heatmap R package. In addition, we also performed Kyoto Encyclopedia of Genes and Genomes (KEGG) pathway enrichment analyses for DEGs using Database for Annotation, Visualization and Integrated Discovery (DAVID), and $P < 0.05$ was considered statistically significant. The top 10 most gene-enriched and statistically significant KEGG pathways were selected for visualization. Finally, we extracted and imported the gene cluster of the most gene-enriched and statistically significant KEGG pathway into the Cytoscape software to analyze the relationships among genes [1]. We ran cytoHubba, a plugin of Cytoscape, to calculate the betweenness value of each gene in the gene cluster, and screened the key gene, which was defined as a gene has largest betweenness value [2].

Supplemental References

1. Shannon P, Markiel A, Ozier O, Baliga NS, Wang JT, Ramage D, et al. Cytoscape: a software environment for integrated models of biomolecular interaction networks. Genome Res. 2003; 13: 2498-504.

2. Chin CH, Chen SH, Wu HH, Ho CW, Ko MT, Lin CY. cytoHubba: identifying hub objects and sub-networks from complex interactome. BMC Syst Biol. 2014; 8 Suppl 4: S11.
